# Fast and automatic assessment of fall risk by coupling machine learning algorithms with a depth camera to monitor simple balance tasks

**DOI:** 10.1186/s12984-019-0532-x

**Published:** 2019-06-11

**Authors:** Amandine Dubois, Audrey Mouthon, Ranjith Steve Sivagnanaselvam, Jean-Pierre Bresciani

**Affiliations:** 1 Department of Neurosciences & Movement Sciences, University of Fribourg, Fribourg, 1700 Switzerland; 2 Grenoble Alpes University, CNRS, LPNC UMR 5105, Grenoble, F-38000 France

**Keywords:** Balance analysis, Fall prevention, Elderly people, Depth camera

## Abstract

**Background:**

Falls in the elderly constitute a major health issue associated to population ageing. Current clinical tests evaluating fall risk mostly consist in assessing balance abilities. The devices used for these tests can be expensive or inconvenient to set up. We investigated whether, how and to which extent fall risk could be assessed using a low cost ambient sensor to monitor balance tasks.

**Method:**

Eighty four participants, forty of which were 65 or older, performed eight simple balance tasks in front of a Microsoft Kinect sensor. Custom-made algorithms coupled to the Kinect sensor were used to automatically extract body configuration parameters such as body centroid and dispersion. Participants were then classified in two groups using a clustering method. The clusters were formed based on the parameters measured by the sensor for each balance task. For each participant, fall risk was independently assessed using known risk factors as age and average physical activity, as well as the participant’s performance on the Timed Up and Go clinical test.

**Results:**

Standing with a normal stance and the eyes closed on a foam pad, and standing with a narrow stance and the eyes closed on regular ground were the two balance tasks for which the classification’s outcome best matched fall risk as assessed by the three known risk factors. Standing on a foam pad with eyes closed was the task driving to the most robust results.

**Conclusion:**

Our method constitutes a simple, fast, and reliable way to assess fall risk more often with elderly people. Importantly, this method requires very little space, time and equipment, so that it could be easily and frequently used by a large number of health professionals, and in particular by family physicians. Therefore, we believe that the use of this method would substantially contribute to improve fall prevention.

Trial registration: CER-VD 2015-00035. Registered 7 December 2015.

## Introduction

Falls in the elderly represent a human, economic and social issue. Indeed, 32-42% of individuals over 70 have already fallen, and these falls often have calamitous consequences [1]. Therefore, reducing and preventing fall risk constitutes a critical issue, now and for the years to come. More frequent assessments of balance abilities and fall risk would allow health professionals to detect at-risk individuals earlier. When provided with appropriate tools and methods, family physicians could be a leading force of this early screening process. They could then direct at-risk individuals towards specialized clinicians, who could perform further assessments, and when required, propose adapted reeducation programs, thereby reducing functional decline, injuries, hospitalizations and placements in retirement homes [2].

Currently, fall risk is often evaluated by health professionals who assess balance abilities [3]. Balance assessments consist of clinical tests such as the Tinetti test (balance and mobility tests) [4] or the Berg Balance test (static and dynamic balance tests) [5]. These tests rely on a visual evaluation of the quality of performed movements and on answers to questions as ’Is the person able or not to hold 15 s on one foot’. The results of these tests are then used to classify the tested individuals as having a high vs low risk of fall. In the literature, a Tinetti score of 36 or less has been shown to identify fallers with a 70% sensitivity and a 52% specificity [6]. Regarding the Berg balance test, Shumway-Cook *et al* [7] demonstrated that a Berg score of 49 or less grants a 77% sensitivity and a 86% specificity. More quantitative, accurate, and objective assessments of postural control can improve the appraisal of balance abilities. For instance, some authors used force platforms to investigate fall risk using posturography ([8], [9]). Hewson *et al* [10] notably observed that in elderly fallers, the center of pressure moves faster than in elderly non fallers. However, quantitative assessment of balance is rarely possible in the clinical practice because equipment such as force platforms or three dimensional movement analysis systems based on cameras (3DMA) is relatively advanced and expensive.

New technologies bring new possibilities, and recently, researchers proposed inexpensive technical solutions to quantify balance. For instance, the Nintendo Wii Balance Board was proposed as potential substitute for force platforms. Similarly, the Microsoft Kinect sensor was suggested as a solution to overcome the cost and time constraint associated to the use of 3DMA systems (e.g., to position the different cameras in the room and markers on the body). In line with this, several studies compared the accuracy of the Kinect to 3DMA systems. For instance, Yang *et al* [11] compared the Kinect and the Optotrack system to extract the center of mass. They showed that both systems were excellent and had comparable test-retest reliability (intraclass correlation coefficient (ICC) > 0.75). In addition, the position variability and average velocity of the center of mass in the horizontal plane showed excellent concurrent validity (ICC > 0.88), and the authors observed a significant linear relationship between the two systems (p < 0.001, r > 0.930). Clark *et al* [12] found an excellent validity (r > 0.75) between the Kinect and the Vicon system for measuring trunk angles. Similarly, Lim *et al* [13] compared the center of mass obtained with two Kinects and with the Vicon system. The two systems provided similar results when measuring changes in the center of body mass (p > 0.05), and the Pearson’s correlation coefficient was relatively large (*γ* > 0.60). The Kinect was also coupled to a Wii Balance Board and compared to a 3DMA system coupled to a force platform [14].

Another line of research consisted in testing whether low cost systems could be used to develop training programs and improve balance abilities in elderly people. For instance, Young *et al* [15] proposed an interface that allows users to calculate the center of pressure of participants standing on a Wii Balance Board and incorporate it into a virtual environment. Lange *et al* [16] developed a tool based on the Kinect for balance training in neurorehabilitation. This study constituted a preliminary exploration of the training based on the low-cost technology without presenting quantitative results. Pisan *et al* [17] found that Kinect-based balance training increases adherence to the exercise. Low-cost technologies can also be used to assess fall risk in elderly people using postural control measurements. Howcroft *et al* [18] used two Wii Balance Boards and were able to identify differences between fallers and non-fallers.

Here we investigated whether balance measurements performed with a low-cost and ’easy-to-set-up’ depth camera could be used to assess fall risk. The balance tasks were chosen because they required little space, little time, and little equipment to be performed. The underlying idea was that coupled to the depth camera and to our machine learning algorithms, these balance tasks could be easily and quickly used by family physicians during their routine check. In order to identify which balance task(s) was/were the most relevant for an early assessment of fall risk, we analyzed the relation between identified fall risk factors and balance performance as quantified using the Microsoft Kinect sensor. Participants taking part in the study had different levels of fall risk, as estimated using 1. known risk factors, namely age and volume of regular physical activity, and 2. performance on the Timed Up and Go (TUG) clinical test. Specifically, muscle loss increases with age and inactivity, which constitute two of the main fall risk factors ([19], [20], [21]). As a consequence, balance control is usually impaired even in healthy and active elderly people [22], even though to a lesser extent than in physically inactive elderly people. The volume of regular physical activity was evaluated using a specific questionnaire, namely the QAPPA questionnaire (see Methods section for details). Fall risk was also assessed using the TUG clinical test. In this test, the evaluated person starts in a sitting position. The person must get up, walk three meters, turn around, come back to the chair and sit down. If more than 13.5 s are needed to perform the test, the person is considered as having a risk of fall. Shumway-Cook *et al* [23] found that a cut-off value of 13.5 s resulted in a discrimination sensitivity of 80% and a discrimination specificity of 100%. We chose this test to assess fall risk with the participants included in our study because this test is widely used by healthcare professionals, and it is recommended by both the American Geriatrics Society and the British Geriatric Society [2]. All participants performed different balance tasks (such as standing on one vs two feet, eyes closed vs eyes open, etc) in front of the depth sensor. Machine learning algorithms were used to determine which balance task(s) and which balance parameters are the more relevant to assess early fall risk.

## Methods

### Participants

Two different age groups participated in the experiment: forty four young individuals (thirty five women, nine men) aged 21 to 29 (mean ± SD = 24.5 ±2.4) and forty older participants (twenty five women, fifteen men) aged 65 to 85 (mean ± SD = 72.9 ±5.2). The main inclusion criteria was being aged 20 to 35 years old for young participants, and 60 to 85 years old for the older participants. In addition, participants should not have fallen in the two years preceding the study. Individuals suffering from orthopedic problem were excluded. On the other hand, participants using auxiliary means to ambulate were included, except if they required a wheelchair. Moreover, none of the young or elderly participants declared any physical impairment nor vision-related issue. The study was conducted in accordance with the Declaration of Helsinki and approved by the local ethics committee.

### Experimental protocol

Three different types of assessments were conducted: a questionnaire-based assessment of physical activity, a balance assessment based on eight balance tasks, and a fall risk assessment based on the TUG test. The three types of assessment are described in detail below. Machine learning algorithms and statistical analyses were used to put in relation the recorded data in the balance tasks with two fall risk predictors, namely the age of the participants and their volume of physical activity, as well as with their performance on the TUG test.

#### Physical activity assessment

The volume of regular physical activity was estimated through the French questionnaire, ’Questionnaire d’activité physique pour les personnes âgées (QAPPA)’, which was validated by De Souto Barreto and Ferrandez [24]. This questionnaire was administered at the beginning of the experiment.

#### Fall risk assessment - TUG test

The TUG test is one of the main reference tests used in clinical environments to assess the fall risk in elderly people. It has been introduced by Podsiadlo and Richardson [25]. In this test, the participant is asked to stand up from a standard chair with arms (after a signal given by the clinical staff), to walk 3 m, to perform a 180 deg turn (in our study, a mark was placed on the ground to indicate to the participants where they had to turn around), to walk back to the chair and to sit down. In our study, participants who performed the test in less than 13.5 seconds (threshold usually considered [23]) were considered as having a low/no risk of fall, whereas participants who needed 13.5 seconds or more were considered as having a high risk of fall. Each participant performed the test three times.

#### Balance assessment - Balance tasks

Balance abilities were assessed using eight different balance tasks. These tasks are part of clinical tests often performed by health professionals to assess balance and the risk of fall recurrence, namely the Berg test [5], the Tinetti test [4] and the Clinical Test of Sensory Interaction and Balance (CTSIB) [26]. The eight tasks are presented in Table 1. The order of presentation of the tasks was counterbalanced. Each of the eight tasks was performed twice with a 5-minute rest period between the two sessions to minimize the effect of fatigue. Some tasks, such as standing on one leg on a foam pad or maintaining a tandem stance (i.e., one foot in front of the other) were particularly difficult for elderly people. Participants experiencing difficulties were allowed to get back to a normal posture during the task. However, the time spent in a ’normal’ posture was counted as time during which the participant was not performing the task adequately. In other words, the considered task duration was the same for all participants: it started when the participant started doing the task, and stopped when the time ’allotted’ for the task elapsed.

**Table 1:** Balance tasks description with their origin and their duration

Number	Origin	Position Feet	Eyes	Duration
1	Berg test, CTSIB	Feet slightly apart	Open	2 min
2	Berg test, CTSIB	Feet slightly apart	Closed	1 min
3	Berg and Tinetti test	Narrow stance	Open	1 min
4	Tinetti test	Narrow stance	Closed	1 min
5	Berg test	On one leg	Open	1 min
6	Berg test	Tandem: one foot directly in front of the other	Open	1 min
7	CTSIB	On two feet, on a foam pad (Airex)	Open	1 min
8	CTSIB	On two feet, on a foam pad (Airex)	Closed	1 min

### Data acquisition and preprocessing

#### Physical activity

We used the QAPPA questionnaire to estimate the time spent practicing physical activity of moderate and vigorous intensity during the seven days preceding the experiment (i.e., number of sessions and average time per session). For each participant, the total amount of time weekly spent to practice physical activity was expressed in MET-min/wee [24]. METs, or metabolic equivalents, are used to describe the energy expenditure of an activity. METs correspond to the ratio between the energy expended during a specific activity and the energy expenditure at rest. The energy expenditure at rest is defined as 1 MET. MET-min/week represent the volume of physical activity per week, and they are calculated by summing up the metabolic equivalent levels of specific activities, taking into account the minutes spent for each activity every week.

#### TUG test

The TUG test was monitored with a Microsoft Kinect v2 sensor. Participants walked perpendicularly to and at a distance of 4.20 m from the Kinect sensor. The TUG was timed using an algorithm providing measurements that are comparable to those performed by health professionals [27]. Performance was measured by averaging the time of the last two trials. The first trial was a familiarization trial, that also allowed us to make sure that the instructions were correctly understood by participants.

#### Balance task

**Fig. 1:**
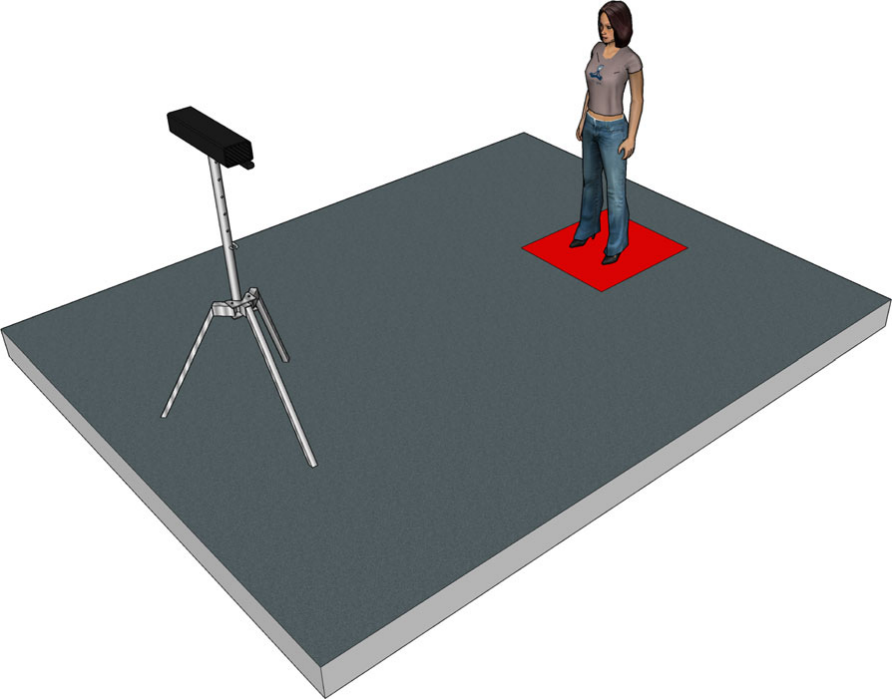
Representation of the experimental set-up with the sensor positioned in front of the participant

For balance tasks, the Kinect sensor was positioned in front of the participants at a distance of 2 m, as illustrated in Fig. 1. Our processing algorithm analyzed the depth images provided by the sensor, and the silhouette of the individuals was extracted using the background substraction method presented in Dubois and Charpillet [28]. To assess balance abilities, the centroid and the body dispersion were extracted from the silhouette. The centroid indicated if the person was stable or not during the task. It is a parameter often used when assessing balance abilities with a camera sensor ([13], [14]). The body dispersion provided information regarding the way participants used their arms to help them maintain balance. Dispersion was smaller when the arms were kept along the body and larger when the arms were moving. The centroid of the person was computed as the average of all points belonging to the silhouette. Body dispersion was calculated as the horizontal dispersion of the pixels cloud. Three parameters were extracted from the centroid and body dispersion:

- variability of the horizontal centroid displacement calculated as the standard deviation of the centroid position on the horizontal plane;
- maximum speed of the horizontal centroid displacement calculated as the maximum of the derivate of the centroid position on the horizontal plane;
- maximum body dispersion calculated as the ratio between the first eigen value and the second eigen value of the covariance matrix.

Participants performed each task twice, so that two values by parameter and by task were obtained for each participant.

### Data analysis

We used unsupervised machine learning methods to cluster the participants in two groups. Specifically, we used the scikit-learn implementation of the K-Means algorithm. This algorithm iteratively updates the clusters’ centroids until their position is stable over successive iterations. In our case, we defined *K*=2 because we wanted to classify participants in two clusters. For each balance task, the K-Means algorithm generated two clusters based on the three standardized parameters of silhouette and dispersion mentioned above, namely maximum speed of the centroid, centroid variability, and body dispersion. For each of the eight tasks, the clustering results were then evaluated taking into account actual fall risk as estimated by 1. risk factors, and 2. TUG performance. Regarding risk factors, we quantified to which extent the two clusters formed by the algorithm were in agreement with the age and volume of weekly physical activity of the participants. Note that the cluster including all young participants was always considered as the ’better balance / lower fall risk’ group. Indeed, all young participants, even those having a low volume of physical activity, had good balance abilities, and none was at risk of fall (the ’worst’ TUG performance for a young participant was 11.17 seconds). Regarding TUG performance, we considered it as being the ’ground truth’ regarding fall risk. Accordingly, average TUG performance (i.e., time) was systematically used as dependent variable to compare the two clusters formed by the algorithm. The comparisons between clusters were performed using Wilcoxon Rank Sum tests, and the significance threshold (i.e., alpha) was set at 0.05. Note that for this statistical analysis, only the elderly people were included in the analysis, because also including the young participants would have ’artificially’ boosted the differences.

## Results

### Clustering analysis

**Fig. 2:**
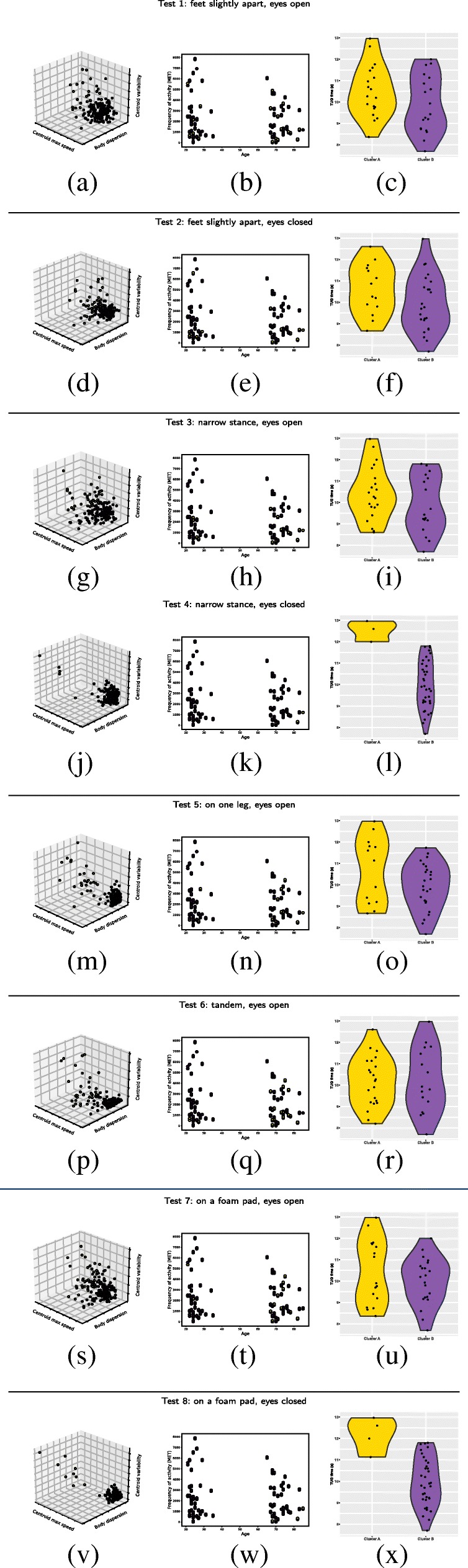
Figure a, d, g, j, m, p, s and v: For each balance task, the K-means clustering method was used to cluster participants in two groups (cluster A for yellow dots and cluster B for purple dots) based on three standardized parameters of silhouette and dispersion (see the three axes). Figure b, e, h, k, n, q, t and w: the outcome of the K-means clustering methods based on the ’Maximum speed of the centroid’ parameter is plotted as a function of the age and volume of physical activity of the participants. The dot color discriminates the two clusters A vs B (yellow vs purple). Figure c, f, i, l, o, r, u and x: Time required to perform the TUG test for the cluster A (yellow) and B (purple). Clusters A and B were formed using the ’Maximum speed of the centroid’ parameter, and only elderly people are represented here

For each balance task, we used the K-Means algorithm to generate two clusters based either on the combination of the three parameters or on each one of the parameters taken individually. This allowed us to determine if single parameters could give rise to a relevant clustering, and if yes, which was / were the most appropriate. The clustering outcome for each balance task based on the three parameters is shown in Fig. 2 (Figure a, d, g, j, m, p, s and v). As highlighted by the graphical representation, the ’separation’ between the two clusters formed by the clustering algorithm is more or less clear-cut depending on the balance task. Specifically, the separation between the two clusters is much clearer for balance tasks 4, 5 and 8 (Fig. 2j, m, v) than for the other balance tasks.

As mentioned above, all young participants had good balance abilities, and none was at risk of fall. Based on this, one prerequisite to consider a model as relevant was that all young participants should have been clustered in the same group. When clustering was based on the combination of the three parameters, of all balance tasks, task 8 was the only one for which all young participants were classified in the same cluster. When only one of the three parameters was considered, irrespective of which one (i.e., all three parameters gave rise to the same outcome), task 8 was once again giving rise to a model regrouping all young participants in the same cluster. Note that for balance task 4, using the ’Maximum speed of the centroid’ by itself classified all young participants in the same cluster. Surprisingly, the latter clustering was ’better’ (for this balance task) than the one in which the three parameters were combined. This constituted the only occurrence of better clustering with only one rather than with three parameters. Overall, using the ’Maximum speed of the centroid’ parameter with tasks 4 and 8 constituted the best simple solution to obtain a relevant clustering in which all the young participants were classified in the same cluster. The results are summarized in Table 2.

**Table 2:** Results of the K-Means algorithm for each task with one or three parameters among ’Maximum speed of the centroid’ (Centroid max speed), ’Maximum body dispersion’ (Body dispersion) and ’Variability of the horizontal centroid’ (Centroid variability). The model considered as relevant was the one with which all elderly participants were clustered in the same group. The table presents the number of young participants in cluster A and cluster B (cluster A - cluster B)

Tasks	Centroid variability	Centroid max speed	Body dispersion	3 parameters
1	5 - 39	16 - 28	31 - 13	38 - 6
2	7 - 37	15 - 29	22 - 22	19 - 25
3	31 - 13	11 - 33	31 - 13	10 - 34
4	10 - 34	**0 - 44**	3 - 41	1 - 43
5	2 - 42	3 - 41	3 - 41	3 - 41
6	1 - 43	3 - 41	1 - 43	1 - 43
7	5 - 39	4 - 40	9 - 35	35 - 9
8	**0 - 44**	**0 - 44**	**0 - 44**	**0 - 44**

**Fig. 3:**
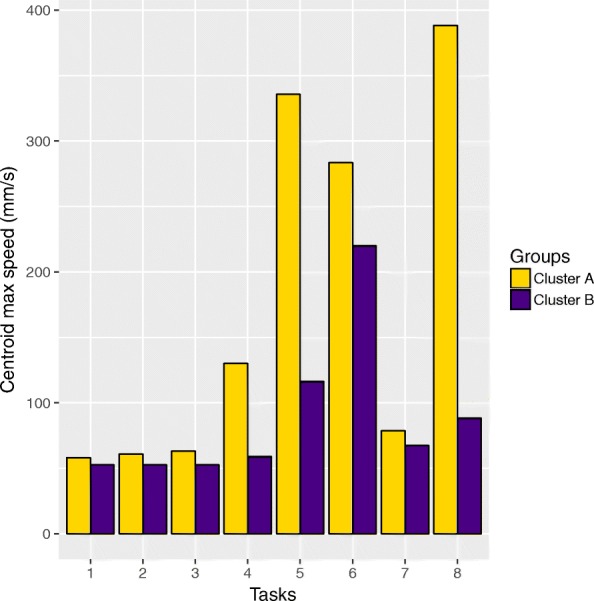
Power of each of the 8 balance tasks to discriminate the two clusters of participants formed by the K-means clustering method with the data of balance task 8, which constitutes the reference here. The purple bars correspond to the cluster B, and the yellow bars to the cluster A

Taking the two clusters formed by the K-means method on balance task 8 as the ’reference’ partition between elderly participants (see previous paragraph), we assessed which of the other seven balance tasks gave rise to the largest differences between these two very clusters. Note that we chose task 8 over task 4 as a reference because for task 8, the clustering outcome was more ’robust’, i.e., the same outcome was obtained whether using one or three parameters. The results are presented in Fig. 3. Tasks 4 and 5, and to a lesser extent task 6, were those leading to the largest difference between the two clusters. Task 6 tended to be difficult for the participants of the two groups. On the other hand, tasks 1, 2, 3 and 7 did not have any discriminative power, and tended to be easy for all participants, irrespective of the cluster they belonged to.

### Relation with age and activity

For each balance task, the outcome of the clustering based on the ’Maximum speed of the centroid’ parameter was put in relation with the age and volume of physical activity of the participants, as shown in Fig. 2 (Figure b, e, h, k, n, q, t and w). We can see that the two clusters formed for balance tasks 4 and 8 are the most relevant in light of these two risk factors. Specifically, for these two balance tasks, the ’yellow’ cluster (cluster A) is constituted of old / very old participants having very little physical activity. The difference between the two clusters was confirmed by statistical analyses. For each task, we used a Wilcoxon Rank Sum test to compare the age and the volume of physical activity per week of the two formed clusters. As shown in Table 3, significant differences in age and volume of physical activity per week between the two clusters were observed for balance task 4 (activity: *p*=0.045, age: *p*=0.014) and balance task 8 (activity: *p*=0.010, age: *p*=0.019).

**Table 3:** Statistically significant differences when comparing age and volume of physical activity per week between the two clusters formed for each task. These comparisons were done using Wilcoxon Rank Sum tests, and the significance threshold (i.e., alpha) was set at 0.05

Variables	Task 1	Task 2	Task 3	Task 4	Task 5	Task 6	Task 7	Task 8
Activity	*p*=0.147	*p*=0.488	*p*=0.568	*p*=0.045	*p*=0.113	*p*=0.493	*p*=0.167	*p*=0.010
Age	*p*=0.364	*p*=0.493	*p*=0.005	*p*=0.014	*p*=0.0004	*p*=0.000009	*p*=0.0002	*p*=0.019

**Fig. 4:**
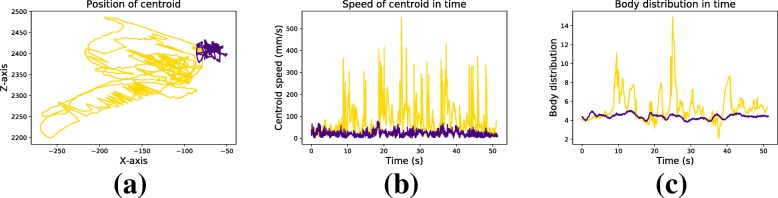
Representation of the three balance parameters (panels a-c) on balance task 8 for two ’typical’ participants. The purple line corresponds to a 83 years old participant of cluster B. This participant walks 90 to 120 minutes every day. The yellow line corresponds to a 82 years old participant of cluster A. This participant has two 30-minute walking sessions per week

Figure 4 illustrates how each of the three balance parameters differ between two ’typical’ participants. These two participants have been put in two different clusters by the K-means clustering method after performing balance task 8. The two participants have the same age (83 vs 82) but a different volume of physical activity (90-120 minutes per day vs 60 minutes per week). The figure illustrates how the participant with a lower volume of physical activity (yellow line) produced more, larger and faster body movements as compared to his more active counterpart (purple line). This is characterized by a higher variability and speed of the centroid (Fig. 4a and b), as well as by larger arm movements (Fig. 4c).

### Relation with the TUG test

The TUG test is a quantitative evaluation of fall risk which is classically used in clinical practice. Therefore, it can be considered as a quantitative ground truth regarding the fall risk status of the participants. Note that though the outcome of the TUG test is usually interpreted relative to a threshold, we considered here that fall risk can also be measured as a spectrum, and that the longer an individual needs to perform the test, the higher his/her objective fall risk is (though we don’t claim that this relation is necessarily linear). For each balance task, the outcome of the clustering based on the ’Maximum speed of the centroid’ parameter was put in relation with the time required to perform the TUG test, as shown in Figure 2 (c, f, i, l, o, r, u and x). As previously mentioned, for each balance task, the K-means algorithm clustered the participants in two groups. For each task, we used a Wilcoxon Rank Sum test to compare the average TUG performance of the two groups, but only taking into account the elderly participants (as including the young participants would have artificially boosted the difference). Significant differences between the two clusters were observed only for balance task 4 (*p*=0.00487) and balance task 8 (*p*=0.00371). As shown in Fig. 2l and x, the participants classified in the cluster B performed the TUG test significantly faster (for test 4: mean performance = 9.99 ms +/- 1.13 and for test 8: mean performance = 9.95 ms +/- 1.13) than the participants classified in the cluster A (for test 4: mean performance = 12.52 ms +/- 0.49 and for test 8: mean performance = 12.17 ms +/- 0.80). Note that for both balance task (4 and 8), all young participants were classified in cluster B. As mentioned above, the TUG performance of the young participants was not included in the analysis, but this highlights the consistency of the clustering method regarding TUG test performance.

## Discussion

Young and elderly participants performed balance tasks in front of a Kinect sensor. Custom-made image processing algorithms automatically extracted the centroid and body dispersion from the recorded silhouette. For each balance task, an unsupervised machine learning algorithm clustered the participants in two groups. The young participants constituted a reference for the clustering algorithm. This step allowed us to identify elderly people with good balance (vs elderly people with ’moderate’ balance performance) ([29–32]). To assess the ’relevance’ of the clustering, the two groups were put in relation with two known factors of fall risk, namely the age and volume of physical activity of the participants ([19–21]), as well as with the performance of the participants on the TUG clinical test. The most relevant and robust balance parameter (when taken in isolation) was the ’maximum speed of centroid’. Using this parameter for the clustering, the two most relevant balance tasks to assess fall risk were the one in which participants had to stand with a normal stance and the eyes closed on a foam pad (task 8), and the one in which participants had to stand with a narrow stance and the eyes closed on regular ground (task 4). Specifically, with these two balance tasks, all young participants were classified in a single group (that we therefore considered as the group with a ’lower fall risk’). We expected this outcome because all young participants taking part in our study had a low fall risk. In that respect, this outcome was actually an important criterion to ’validate’ our classification. In addition, with these two tasks, the elderly people that were classified in the ’higher fall risk’ group (i.e., the group without any young participant in it) were the oldest and/or the least active participants. Finally, for these two tasks, there was a significant difference between the average TUG performance of the two clusters. Specifically, the elderly participants that were in the ‘higher fall risk’ group performed the TUG test significantly slower than the elderly participants that were classified in the other group (that was considered as the ‘lower fall risk’ group). Taken together, these results show that for the two above-mentioned balance tasks, coupling our machine learning algorithm to a depth sensor allowed us to automatically classify elderly participants according to their fall risk, as estimated using known factors such as age, level of physical activity, and time on the TUG test.

In the literature, centroid oscillations often constitute the parameter of choice when assessing balance abilities with a camera sensor ([13, 14]). Here, we measured an additional balance-related parameter, namely ’Maximum body dispersion’. This is because we wanted to gather some additional information relative to the ’balance strategy’ used by participants. In particular, we wanted to know whether they needed to use their arms to maintain balance. We observed that the clustering based on the ’Maximum body dispersion’ parameter was relevant only for task 8, i.e., the task in which participants had to stand with eyes closed on a foam pad. Note that for this task, the ’Maximum body dispersion’ parameter gave rise to the same clustering as the centroid-related parameters. As mentioned above, the most relevant balance parameter was the ’maximum speed of centroid’, because it provided a relevant model both for balance task 4 and balance task 8. Taken together, our results suggest that measuring the ’maximum speed of centroid’ is necessary and probably sufficient to assess fall risk in the elderly, provided the measurements are made on relevant balance tasks, namely standing with a normal stance and the eyes closed on a foam pad and standing with a narrow stance and the eyes closed on regular ground.

All eight balance tasks used in this study were chosen because they are included in the clinical tests routinely used by healthcare professional to assess fall risk in patients. These clinical tests might be burdensome and are usually performed only when some risk has already been identified. For this reason, we tested here whether simple balance tasks monitored by a depth sensor could efficiently assess fall risk in the elderly. Our results suggest that some balance tasks are less discriminating, because they were performed without any problem by all elderly participants, irrespective of their actual fall risk. This was notably the case for the tasks requiring to stand feet slightly apart (with eyes open or closed), to stand with a narrow stance and the eyes open, or to stand on a foam pad with the eyes open, namely tasks 1, 2, 3 and 7, respectively. On the other hand, the balance task requiring the participants to stand with a tandem stance (i.e., one foot directly in front of the other, task 6) was particularly difficult for all elderly participants. The difficulty of this task probably relates to the peculiarity of the required position, which is neither natural nor frequently used, unlike other positions like standing static on a foot to get dressed or keep your balance on a slightly unstable ground. Ultimately, the balance tasks that gave rise to the most relevant clustering were the ones requiring the participants to stand with the eyes closed, either with a narrow stance on regular ground or with a normal stance on a foam pad (task 4 and task 8). Indeed, these balance tasks were the ones that best discriminated elderly participants according to their fall risk. It is interesting to note that out of the eight tasks proposed to the participants, these two were the only ones combining two ’difficulties’. Specifically, the participants were deprived of visual information and required to adopt an unstable stance. These constraints forced the participants to rely more on kinesthetic and vestibular information. Gadkaree *et al* [33] showed that 70 to 79 year old individuals having dual or triple sensory impairment are characterized by poor physical performance, which is often associated to low levels of physical activity. Therefore, it seems logical that in our study, the active elderly participants were the ones who fared the best in balance tasks 4 and 8.

In this article, we show that fall risk can be quickly and reliably assessed by using a low cost sensor to measure the maximal centroid speed during simple balance tasks such as standing with the eyes closed, either with a narrow stance or on a foam pad. Even though none of the elderly participants that were included in our study was currently considered at risk of falling (none of them ever fell and all performed the TUG test in less than 13.5 s), our method identified the participants for which fall risk was the highest according to their age, their volume of physical activity, and their TUG performance. One of the advantages of our system is that it would allow clinicians to target elderly people at higher fall risk (based on risk factors such as age and/or physical activity) without having to carry out a questionnaire or different clinical tests. This would constitute a very important prevention step, because most of the time, clinical tests are performed only after the occurrence of the first fall. Here the practitioner would only need to ask the person to stand for 1 minute with the eyes closed on a foam pad or with a narrow stance in front of the Kinect sensor. It is quick, easy, and requires little space. In addition, no particular expertise is required because the system automatically provides the performance and the result of the fall risk assessment without any need for interpretation. For all these reasons, this system could be used with more flexibility and more routinely by a large number of health professionals, which would substantially improve fall prevention. The modest space and time requirements and the ease of use would notably allow general practitioners to effortlessly integrate the procedure to their check up, which is much more complicated with clinical tests such as the TUG. The ease of use would also facilitate a longer follow-up of patients. The main limitation of this study is that it (purposely) focused on individuals having a low to intermediate risk of fall. This is because our goal was to be able to identify early and subtle signs of fall risk in order to improve fall prevention in the future. Future studies will also integrate elderly people who have already fallen, i.e., individuals having a higher fall risk. Along that line, future research will also rely on the system and the balance tasks presented here to perform longitudinal studies in order to follow the evolution of the relation between the clustering results and fall occurrence.

## Conclusion

Currently, fall risk is often evaluated by health professionals who assess balance abilities. Assessment protocols are often subjective and can vary between examiners and clinical settings. In addition, clinical tests might be burdensome and are usually performed only when some risk has already been identified. More quantitative, accurate, and objective assessments of postural control would improve the appraisal of balance abilities. Here, we show that fall risk can be quickly and reliably assessed by coupling a low cost ambient sensor with machine learning algorithms to monitor simple balance tasks such as standing with the eyes closed with a narrow stance or on a foam pad. The system that we propose is quick, easy to use, and it requires little space. Therefore, this system could be used with more flexibility and more routinely by a large number of health professionals, which would substantially improve fall prevention and facilitate a longer follow-up of patients.

## Abbreviations

TUG: Timed Up and Go 3DMA: Three Dimensional Movement Analysis ICC: Intraclass Correlation Coefficient CTSIB: Clinical Test of Sensory Interaction and Balance
